# Nanoparticles: Small Interventions for Major Challenges in Aquaculture

**DOI:** 10.3390/biology15161431

**Published:** 2026-08-19

**Authors:** Laura González-Rodríguez, Patricia Pereiro, Beatriz Novoa, Antonio Figueras

**Affiliations:** Instituto de Investigaciones Marinas (IIM-CSIC), C/Eduardo Cabello, 6, 36208 Vigo, Spain; lgonzalez@iim.csic.es (L.G.-R.); beatriznovoa@iim.csic.es (B.N.)

**Keywords:** nanoparticles, aquaculture, fish health, environmental remediation, consumer safety

## Abstract

Aquaculture has emerged as one of the fastest-growing sectors of global food production, driven by population growth, consumer demand, and technological innovation. Despite this expansion, the industry continues to face persistent challenges, including disease management, optimizing growth and product quality, maintaining water quality, and mitigating environmental impacts. Nanotechnology has recently emerged as a promising tool to tackle these issues, offering both direct applications, such as antimicrobial agents, immunostimulants, and optimized drug delivery to enhance fish health, and indirect applications, including post-harvest processing, environmental monitoring, and contaminant removal. This review provides a comprehensive overview of nanoparticle applications in aquaculture from 2020 to early 2026, covering the various types of nanoparticles, their mechanisms of action, and the specific materials investigated during this period. The discussion also addresses potential benefits, limitations, and biosafety concerns, emphasizing the need for further in vivo and field studies to ensure the safe and sustainable integration of nanotechnologies into aquaculture practices.

## Simple Summary

Aquaculture plays a key role in providing healthy food for a growing global population, but the industry faces major challenges, including infectious diseases, water pollution, and the need to reduce the use of antibiotics and other chemicals. Nanotechnology offers innovative solutions to many of these problems. Tiny materials, known as nanoparticles, can improve the delivery of medicines and vaccines, help remove contaminants from water, detect pathogens at an early stage, and extend the shelf life of seafood products. This review summarizes the latest advances in the use of nanoparticles throughout the aquaculture production chain, from improving fish health and water quality to enhancing food safety after harvest. It also discusses the possible risks associated with their use, including their effects on aquatic organisms, the environment, and consumers, as well as the current need for clear safety regulations. Overall, nanotechnology has great potential to make aquaculture more sustainable, efficient, and environmentally friendly, but further research is needed to ensure that these new materials can be used safely before they are widely adopted by the industry.

## 1. Introduction

Aquaculture has become a major component of global food production and plays an increasingly important role in meeting the growing demand for aquatic foods. In recent decades, the sector has experienced substantial growth, with global aquaculture production of aquatic animals reaching 103 million tonnes in 2024, with a total value of USD 371 billion [1]. Aquaculture now accounts for 53% of global aquatic animal production and over 59% of aquatic animal food output [1]. In Europe, increasing catch limits and restrictions on traditional fishing activities for key commercial species in the Atlantic, North Sea, Mediterranean, and Black Seas are further contributing to the growing importance of aquaculture as a source of aquatic foods [2].

Beyond its economic importance, aquaculture contributes to food security by providing a reliable source of nutritious animal protein and micronutrients [3]. The sector is expected to continue expanding over the coming decade, driven by increasing global demand for aquatic foods associated with population growth and rising per capita consumption [3].

This continued development also creates opportunities to improve the efficiency, sustainability, and quality of aquaculture production. Effective management and technological innovation can contribute to optimizing resource use, reducing waste, improving animal health and welfare, and enhancing the quality and safety of aquatic foods. These objectives are aligned with the FAO Blue Transformation Roadmap 2022–2030, which promotes the sustainable development of aquatic food systems and emphasizes improved productivity, sustainability, inclusiveness, and innovation in aquaculture [1].

As aquaculture production intensifies, several challenges need to be addressed to ensure the long-term sustainability of the sector. These include the management of infectious diseases, the optimization of animal growth and nutritional status, the maintenance of water quality, the reduction in environmental impacts, and the preservation of food quality and safety throughout production and post-harvest processes [4]. Addressing these challenges requires innovative strategies capable of improving production efficiency while minimizing environmental impacts and maintaining the quality and safety of aquatic foods.

Among the emerging technologies being explored for these purposes, nanotechnology has attracted increasing attention. Nanoparticles (NPs) possess distinctive physicochemical properties that can be exploited to develop novel approaches across the aquaculture production chain [5]. In this review, we explore the application of this emerging technology in aquaculture, focusing on its potential to improve three key and interconnected aspects of the sector: fish health and disease management, fish production and food quality, and water quality and environmental sustainability.

### 1.1. Fish Disease Management

Maintaining good health and welfare is essential for efficient and sustainable aquaculture production and for ensuring a consistent supply of high-quality aquatic foods. Infectious diseases caused by bacteria, viruses, parasites, and fungi remain an important factor affecting aquaculture productivity and profitability worldwide. It is estimated that infectious diseases account for the loss of roughly 10% of farmed aquatic animals globally, representing annual economic damages of more than 10 billion USD [6]. These microorganisms are widespread in aquatic environments, often forming a substantial part of the normal water flora, and are responsible for many fish diseases. They typically cause illness only when fish experience stress from various factors.

In this context, it is essential to implement rigorous disease control measures by evaluating all relevant factors, including the presence of environmental pathogens, species-specific susceptibility, and adverse conditions in the aquatic environment, particularly those resulting from the confinement of high-density populations [7]. Current aquaculture practices increasingly combine preventive and therapeutic strategies, including vaccination, improved biosecurity, nutritional interventions, and the targeted use of antimicrobial and antiparasitic treatments. Continued innovation in these areas is essential to improve disease prevention and control while reducing the environmental and economic costs associated with disease outbreaks.

### 1.2. Fish in the Food Industry

In aquaculture, the size and morphological characteristics of individual fish are critical for maintaining reproductive potential and stock stability, which in turn affects the quality of products available for sale. Furthermore, there is a direct correlation between fish size and weight and overall profitability [8,9]. For example, a study by Sjoberg found that it is more advantageous to produce a single fish weighing 2 kg than two fish weighing 1 kg each [10]. Other factors that can influence consumer decisions include freshness and organoleptic properties, such as overall appearance, color, odor, and flavor. These attributes are typically assessed through indicators such as gill condition and the brightness of the fish, among others [11].

In this context, the nutritional status of fish is an important parameter to consider. Nutritional disorders in aquaculture can result from nutrient deficiencies, excessive nutrient intake, or an imbalanced diet [12]. Typically, these alterations develop gradually, and symptoms may not become apparent until later stages. By that time, effective intervention may be difficult, as affected fish can become weakened and more susceptible to opportunistic microbes, potentially leading to chronic diseases. Common indicators of nutritional disorders include reduced fertility, slower growth rates, loss of appetite, increased disease susceptibility, impaired movement, and histopathological abnormalities [13].

Today, aquaculture seeks to innovate in both farming and processing technologies, including improvements in feed formulation, seed and fry production, disease management and biosecurity, as well as fish processing for human consumption, among other areas, to meet consumer and market demands [14]. In particular, food packaging is essential for maintaining the freshness of many common foods and facilitating storage and transportation. It also plays a key role in ensuring food safety, quality, and security in daily life [15].

### 1.3. Water Quality and Environmental Impact

Maintaining the health and well-being of aquaculture animals clearly depends on water quality, which constitutes their natural habitat. Consequently, aquaculture faces unique challenges compared to other animal production systems, including the need for life support systems to maintain water balance and ensure that conditions meet the specific requirements of each species. During the aquaculture cycle, wastes are generated from unused inputs or by-products, including feed, chemicals, drugs, and pathogens. These wastes can be classified into two categories: solid and dissolved. Solid wastes primarily originate from undigested or uneaten feed and are excreted as faeces. They can be further categorized as settled solids, which sink rapidly, or suspended solids, which remain dispersed in the water. On the other hand, dissolved solids include a variety of compounds, such as nitrogen-, copper-, or phosphorus-based substances, which can be toxic to fish at high concentrations [16,17]. The accumulation of nutrients can promote eutrophication processes, leading to excessive algal growth and subsequent oxygen depletion, which may result in hypoxia or even anoxia, threatening fish survival [16,17]. Therefore, these contaminants not only increase the pathogen load in the system but can also act as stressors, weakening the fish’s natural resistance and making them more susceptible to infections [18].

On the other hand, controlling the environmental impact of aquaculture is essential, as it contributes to a broader public concern over aquatic pollution. Although aquaculture is not the primary source, industrial and agricultural runoff, which contains high levels of pesticides and herbicides, also plays a major role. Effective water quality management and remediation are crucial for addressing current wastewater issues. Emphasis should therefore be placed on targeted interventions that improve the overall performance of aquaculture facilities, given the close interconnection between these factors. This involves applying innovative approaches both directly to the entire water treatment process and indirectly through measures such as efficient drug use, enhanced immunity, growth promotion, and effective vaccination in fish, among others [19]. Adopting these strategies also optimizes the economic performance of the aquaculture industry while promoting sustainable practices.

## 2. Nanoscale Technology in Aquaculture

The term ‘nanotechnology’ refers to a branch of science and engineering that focuses on structures at the nanoscale, typically between 1 and 100 nanometers. A major area of research in this field is the development and study of nanoparticles. In nanotechnology, a nanoparticle is a small object whose material exhibits unique properties not observed under normal conditions, such as an increased surface area-to-volume ratio and distinctive physicochemical characteristics. These properties have enabled the creation of innovative devices, tools, and systems with unprecedented precision and efficiency. Consequently, nanotechnology has found applications across numerous fields, including electronics, energy, environmental remediation, and healthcare for humans and animals, among others [16,20].

**Figure 1:**
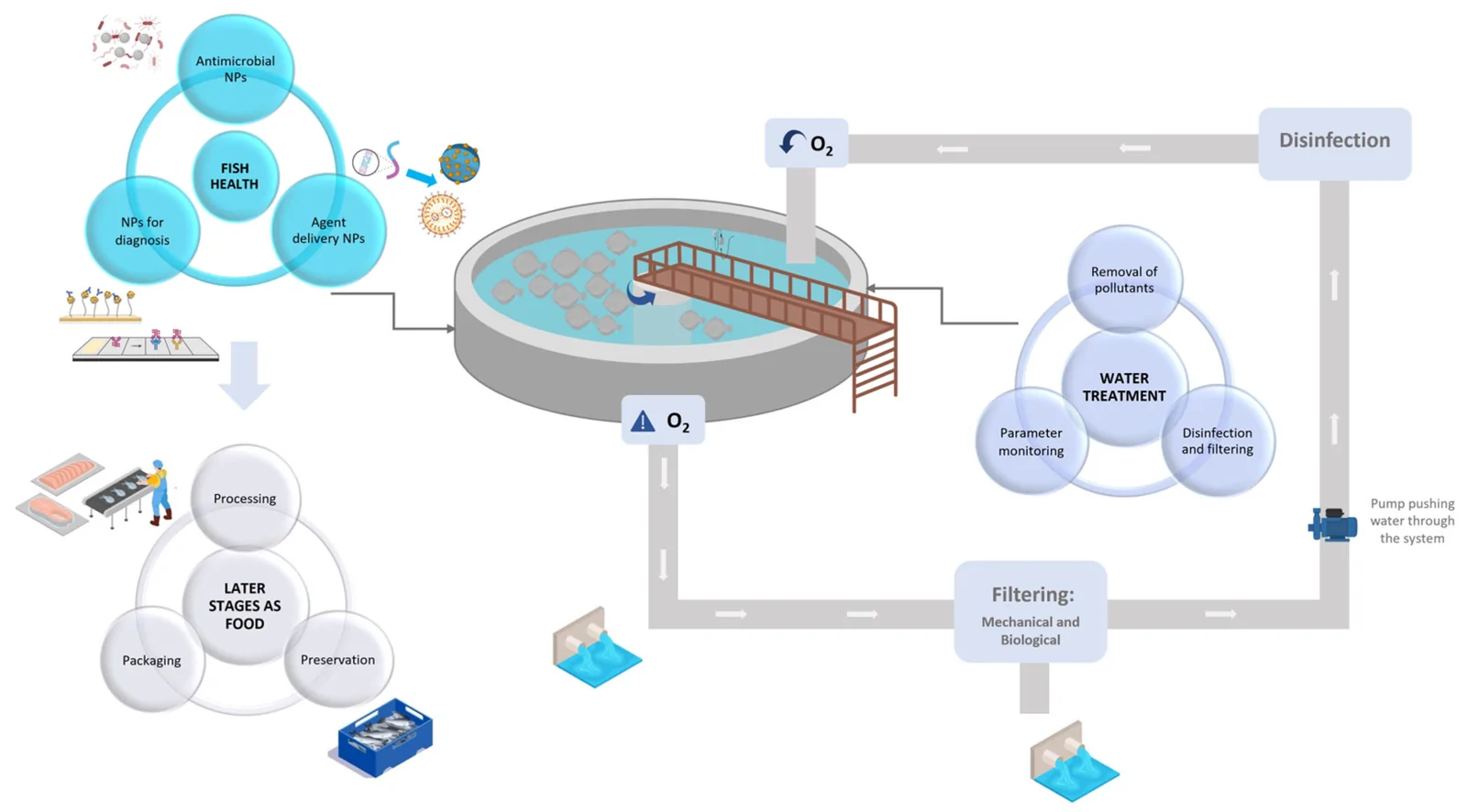
Potential sites of nanoparticle action within a closed-loop aquaculture system. Created in Biorender. Laura González-Rodríguez (2025) (https://biorender.com/).

The aquaculture sector has recently begun using nanoparticles (NPs) to tackle some of the industry’s challenges in an economically and environmentally sustainable manner, although significant potential for further applications still remains. Nanotechnological innovations can have both direct and indirect applications in aquaculture (Figure 1). Direct applications primarily focus on aquatic health management, including the use of feed enhancers, immunostimulants, and optimized drug delivery, with the aim of improving aquatic animal production—enhancing quality, taste, texture, flavour, and nutrient content of fish as food, which is the ultimate goal of the sector. Indirect applications include managing the post-harvest process, monitoring key water and environmental parameters, protecting against pathogens, and removing toxic substances from the environment without negatively affecting the fish [5]. In an aquaculture facility, both direct and indirect interventions can be implemented at the tank level as well as in the effluents. From a broader perspective, any such action represents an improvement. For example, preventing biofilm formation in tanks reduces bacterial proliferation, lowering the risk of fish disease, decreasing the need for intensive antibiotic treatments, and facilitating the handling and processing of final products.

In addition to individual nanoparticles, increasing attention has been directed toward nanocomposites, which combine nanoparticles with polymers, ceramics, carbon-based materials, or other matrices to improve stability, mechanical properties, controlled release, and functionality. In aquaculture, these hybrid materials are being explored for applications including drug delivery, water remediation, biosensing, and food packaging, often providing advantages over single-component nanoparticles.

## 3. Classification of Nanoparticles by Application in Aquaculture

Nanoparticles are commonly categorized based on various factors, including origin, dimensionality, pore size, and composition. However, in this review, we focus on classification according to their potential applications. Below, we present and discuss some of the most significant types of nanoparticles that have been studied since 2020 for possible use in aquaculture, with the aim of addressing key challenges faced by the industry. Although the nanoscale is conventionally defined as ranging from approximately 1 to 100 nm, the terminology used to describe particulate systems is not always consistent across the scientific literature. In particular, particles exceeding 100 nm are also frequently referred to as NPs, despite falling outside the strict nanoscale definition. Therefore, in this review, we considered particulate systems with average sizes of up to approximately 300 nm. This range was selected to encompass the particle sizes commonly described as NPs in the aquaculture literature while excluding clearly micron-sized particulate systems.

### 3.1. Fish Health

#### 3.1.1. Nanoparticles with Direct Antimicrobial Activity: Metal-Based NPs as the Focal Point

While disease outbreaks in aquaculture are increasingly managed using antibiotics and chemotherapeutics, there is growing concern over the consequences of their widespread use. These include drug residues in feed that can enter the food chain and impact human health, the development of antimicrobial resistance in pathogens, and the detrimental effects of these residues on aquatic ecosystems and populations [21]. Although antibiotics remain the farmers’ first choice, alternative and safer methods are available, including the use of NPs with antimicrobial activity. Due to their unique properties, NPs have emerged as effective and promising tools for combating bacterial infections. Their small size allows them to penetrate bacterial membranes easily, while their high surface area-to-volume ratio facilitates interactions with receptors on the bacterial surface [22].

Metallic NPs are the most widely used inorganic nanoparticles, with silver nanoparticles (AgNPs) receiving particular attention. AgNPs have demonstrated significant success as antibacterial agents across a wide range of applications due to their ability to disrupt bacterial cells at multiple levels. Studies suggest that their antibacterial activity involves: (1) alterations in bacterial morphology and structure, which are directly related to the size and shape of the nanoparticles, and (2) damage associated with the release of silver ions following AgNP oxidation, which can contribute to oxidative stress through reactive oxygen species (ROS) generation. Particle size is an important determinant of antimicrobial activity, as smaller AgNPs provide a greater specific surface area and can therefore interact more extensively with bacterial cells, resulting in enhanced antibacterial and anti-biofilm activity compared with larger particles [23,24]. Particle morphology may also influence these effects by modifying nanoparticle-cell membrane interactions and the exposed surface, thereby affecting their reactivity and antimicrobial activity [24]. These size- and shape-dependent interactions may promote membrane disruption, oxidative stress, and cellular damage, ultimately contributing to bacterial death and the inhibition or destabilization of biofilms.

In addition, in most of these cases, green synthesis techniques are used, as they are less expensive, reduce pollution, and enhance environmental and animal safety. These methods utilize a variety of natural sources, including bacteria, fungi, plants, and other biomaterials, to produce nanoparticles in an eco-friendly manner [25]. However, the use of AgNPs in aquaculture and animal husbandry is still relatively new; although some in vitro results are available, in vivo studies remain very limited [26].

AgNPs have been applied to inactivate a range of bacterial fish pathogens, as summarized in Table 1. Studies have focused on the most relevant bacterial genera affecting aquaculture species, including *Vibrio* (notably *V. parahaemolyticus*, *V. harveyi*, and *V. alginolyticus*), *Aeromonas* (particularly *A. hydrophila*), and *Pseudomonas* (*P. putida* and *P. aeruginosa*) [27,28,29,30,31,32,33,34,35,36,37]. Some authors have also investigated bacteria of both veterinary and public health relevance, including species pathogenic to fish, as well as species that may pose a risk to human consumers through post-harvest contamination during processing, handling, or storage.

There are relatively few in vivo studies investigating this topic. Notable examples include Ni et al., observed that the addition of AgNPs to the culture water of zebrafish (*Danio rerio*) for 30 days resulted in a higher growth performance, improved immune parameters, and potential positive modulations on intestinal microbiota [38]; Qi et al., who examined the impact of feeding zebrafish with AgNPs encapped by dihydromyricetin as a therapeutic against methicillin-resistant *Staphylococcus aureus* [39]; Zhang et al., who found that AgNPs synthesized using extracts from the mushroom *Flammulina velutipes* as reductant and stabilizing agents significantly reduced the mortality of the Manila clam (*Ruditapes philippinarum*) infected with *V. parahaemolyticus* [34]; and Aly et al., who evaluated the effects of feeding AgNPs to Nile tilapia (*Oreochromis niloticus*) for preventing *A. hydrophila* infection and obtaining positive results [40].

However, several researchers have demonstrated that AgNPs exhibit effects beyond their antibacterial activity, also impacting non-bacterial pathogens that affect humans and other animals [41].

The toxicity of AgNPs to common aquaculture parasites, including the crustacean ectoparasite *Argulus siamensis*, the trematodes *Prohemistomum*, *Clinostomum* and *Euclinostomum*, and the ciliated protozoa *Tetrahymena*, has been investigated in recent years [42,43,44]. In the monogenean *Cichlidogyrus*, Pimentel-Acosta et al. even report the expression profiles of genes involved in key processes that are dysregulated by AgNPs [45]. They all support the idea that the parasite’s toxicity and nanoparticle size are negatively correlated.

To the best of our knowledge, the only efficacy studies using AgNPs (since 2020) have targeted spring viremia of carp virus (SVCV), European catfish virus (ECV), and Ictalurid herpesvirus 2 (IcHV-2) [46]—all studied in vitro—as well as a non-fish aquaculture virus, white spot syndrome virus (WSSV), which has been studied both in vitro and in vivo and with the AgNPs showing positive effects [47].

Nevertheless, despite the strong efficacy of AgNPs against a wide range of bacteria, the release and accumulation of silver-based antimicrobial agents in the environment are increasing with their extensive use, leading to a growing prevalence of silver-resistant bacteria [48]. Evidence suggests that bacterial resistance to silver may arise through multiple mechanisms, including the promotion of AgNP aggregation, reduced interaction between silver and bacterial cells, enhanced reduction in Ag^+^ ions, and increased efflux of silver from the cell [48]. Therefore, just as has been the case with antibiotics for decades, we must also exercise caution in the use of alternative antimicrobial strategies, as bacteria have an extraordinary ability to adapt to new therapeutic approaches.

The direct antimicrobial potential of other NP types has also been investigated against aquaculture pathogens, as summarized in Table 1. Among the inorganic nanoparticles, metal-based NPs such as zinc (Zn) [30,49,50,51,52], copper (Cu) [53,54], gold (Au) [55,56], and selenium (Se) [43] have been studied. In addition, chitosan (CS) has been used as a model polymer for organic NPs [43,57,58].

Overall, metal-based nanoparticles show considerable antimicrobial activity against a broad range of aquatic pathogens, but their direct application as antimicrobial agents in aquaculture remains largely experimental. The strong antimicrobial effects reported in vitro do not necessarily translate into equivalent efficacy under farming conditions, where nanoparticle behaviour is affected by water chemistry, organic matter, and interactions with other components of the production system. In addition, potential toxicity to cultured animals and non-target organisms, environmental persistence and accumulation [59], and the possibility of selecting resistant microorganisms [48] raise important concerns regarding their repeated or large-scale use. Thus, further in vivo studies under realistic aquaculture conditions are needed to determine whether the antimicrobial benefits of these nanoparticles can be achieved at safe and economically feasible concentrations without generating unacceptable environmental risks.

**Table 1:** Overview of studies on nanoparticle-mediated antimicrobial effects targeting aquaculture pathogens, based on the reviewed literature.

Specific Effects	Type of NP	Reference
In vitro and in vivo assays against marine *V. parahaemolyticus*	Ag	[31,34]
Effective anti-vibriocidal (*V. parahaemolyticus* and *V. harveyi*) and antioxidant properties	Ag	[28,32]
Antibacterial and antibiofilm activity against *A. hydrophila* and *P. aeruginosa*	Ag	[33]
Efficacy against *A. hydrophila* and *Flavobacterium aquidurense*	Ag	[35]
Antimicrobial and antibiofilm activity against *P. aeruginosa*, *Escherichia coli* and *Candida albicans*	Ag	[34]
Effective against *S. agalactiae*, *A. hydrophila* and *V. alginolyticus*	Ag	[36]
Activity against *Plesiomonas shigelloides*, *Pseudomonas alcaligenes* certain species of the genus *Flavobacterium*. The growth of beneficial bacteria is promoted (*Rhodococcus erythropolis*)	Ag	[38]
In vitro evaluation with 7 different bacterial types and in vivo assay that demonstrated that coated AgNPs was more efficient than non-coated ones in reducing intestinal bacterial colonization by *A. hydrophila* in Nile tilapia	Ag	[27]
In vivo assay against *S. aureus* in zebrafish model	Ag	[39]
In vitro and in vivo (in Nile tilapia) antibacterial inhibitory effect against *A. hydrophila*	Ag and CS	[40]
Activity against *P. putida*, *Edwardsiella tarda*, *A. hydrophila* and *Yersinia enterocolitica*	Ag and Zn	[30]
Antibacterial efficacy against *Clostridium perfringes* in Nile tilapia	Zn	[50]
Action against different *Aeromonas* strains, *P. aeruginosa*, *Edwardsiella ictaluri* and *Streptococcus iniae*	Zn	[49]
Activity against *S. aureus* bacterial strain and *C. albicans* fungal strain	ZnO	[51]
Antibacterial effectivity against *S. aureus* and *E. coli* resistant strains	CuO	[53]
Antibacterial activity against gran negative *Vibrio* species	Cu	[54]
Activity against *V. harveyi* and *S. aureus*	Au	[56]
Bactericidal activity against *A. hydrophila* both in vitro and in vivo in Nile tilapia	CS	[58]
Strong antibacterial efficacy was observed against *Pseudomonas fluorescens*, *A. hydrophila* and *Yersinia ruckeri*: moderate activity was recorded against *P. aeruginosa*, *P. putida*, *E. tarda*, *Aeromonas salmonicida* subsp. *salmonicida*; while lower activity was noted against *E. ictaluri*, *Francisella noatunensis* subsp. *orientalis*, *Aeromonas caviae*, *Aeromonas veronii*, and the fungi *Aphanomyces invadans* and *Saprolegnia parasitica*. In contrast, Vibrios (*V. vulnificus*, *V. parahaemolyticus*, *V. harveyi*, *V. alginolyticus*) and *Citrobacter freundii* were highly resistant to CSNP suspensions	CS	[57]
In vitro anti-parasitic efficacy against *Argulus siamensis*	Ag	[42]
In vitro and in vivo antiparasitic assays demonstrating different efficacies depending on the NPs against Clinostomid and *Prohemistomid metacercariae*	Ag, Se and CS	[43]
Antiprotozoal efficacy against *Tetrahymena* sp., without evidence of harmful effects on fish	Ag	[44]
Incorporation of NPs alleviates the effect of *Trichodina* species infection in Nile tilapia. Also demonstrated antioxidant, antibacterial and antifungal activities	Zn	[52]
In vitro antiviral activity against SVCV, ECV and IcHV-2	Ag	[46]
Potential as an antiviral additive in feed against WSSV	Ag	[47]
In vitro activity against WSSV and complementary in silico approach	Au	[55]

#### 3.1.2. Nanoparticles for Agent Delivery

Another approach to disease control -whether infectious or not- is the use of nanoparticles (NPs) for targeted delivery of therapeutic compounds. This strategy minimizes the risk of side effects, enhances therapeutic efficacy, accelerates fish recovery, and reduces environmental impact by decreasing the amount of medication released into the aquatic environment [60]. The therapeutic advantages of these so-called nanocarriers include enhanced solubility and stability, resulting in improved bioavailability of the administered compounds [61]. In this context, a range of compounds, including hormones, vitamins, antibiotics, and vaccines, have been delivered using nanocarriers in aquaculture, aiming not only to treat diseases but also to prevent them, boost immune responses, promote growth, and achieve other health-related benefits.

Due to their remarkable biocompatibility and biodegradability, as well as the relatively simple methods available for material encapsulation, organic nanoparticles, such as polymer- and lipid-based carriers, are among the most widely used nanocarriers in this context. The most common forms of organic nanoparticles are nanospheres and nanocapsules, in which the molecules of interest can either be adsorbed onto the surface or entrapped within the internal matrix [62].

Despite these promising advantages, the translation of nanocarrier-based delivery systems from experimental studies to practical aquaculture applications remains challenging. Their performance depends not only on the biological efficacy of the delivered compound but also on factors such as formulation reproducibility, loading capacity, stability during storage and administration, scalability of the production process, and manufacturing costs [63]. These aspects are particularly relevant in aquaculture, where treatments often need to be produced and administered on a large scale. Therefore, although nanocarriers may improve the efficacy and reduce the environmental impact of conventional therapeutics and vaccines, their commercial applicability will ultimately depend on whether these benefits can be achieved consistently and safely, while maintaining costs compatible with aquaculture production.

##### The Predominant Use of Polymeric NPs: Chitosan

Among polymers with potential applications in aquaculture, chitosan nanoparticles (CSNPs) have gained considerable attention in recent years and are being studied both alone and in combination with other polymers [64]. Chitosan, derived from chitin, a naturally occurring polysaccharide found in the exoskeletons of marine crustaceans, insects, and the cell walls of fungi, is an ideal candidate for encapsulating unstable compounds due to its ability to provide controlled release. In aquaculture, it is particularly suitable for inclusion in diet formulations, as its oral administration does not compromise its biodegradability or biocompatibility [65].

Regarding the use of chitosan as a carrier to support and/or enhance the action of the primary compound, several studies have been conducted in recent years (Table 2). Elnagar et al. (2024) incorporated vitamins C and E into chitosan nanoparticles (CSNPs) and observed an improved response to salt-induced stress and *S. agalactiae* infection in Nile tilapia [66]. Similarly, Ibrahim et al. (2021) reported enhanced immunostimulatory and antioxidant responses when selenium-loaded CSNPs were incorporated into the diet [67]. In line with this, other authors have encapsulated phytochemicals, N-acetyl-D-glucosamine, or immune-related proteins such as IFN-γ2, and have reported enhanced immune and antioxidant responses, along with increased antibacterial activity [68,69,70]. On the other hand, goldfish (*Carassius auratus*), a model species in reproductive biology and aquaculture, exhibited accelerated oogenesis following dietary administration of a GnRH analogue encapsulated in CSNPs [71]. To the best of our knowledge, the only in vitro study reported is that of Fabrikov et al. (2024), which demonstrated that incorporating a specific type of polyphenols into a chitosan–alginate mixture significantly increased the growth inhibition percentage for all pathogenic species tested, with *Photobacterium damselae* showing over 90% growth inhibition in particular [72].

Another potential application of chitosan nanoparticles (CSNPs) in aquaculture is vaccine delivery. By protecting antigens from degradation by bodily enzymes such as nucleases, proteases, and phosphatases, encapsulation has been shown to enhance immunogenicity. Specifically, nanoparticles are preferentially phagocytosed by dendritic cells, leading to antigen presentation via MHC class I and activation of cytotoxic T cell responses. In contrast, microparticles are more likely to be phagocytosed by macrophages, resulting in antigen presentation via MHC class II and a humoral immune response. This difference arises because particle size influences the delivery of the plasmid to specific immune cell types, thereby optimizing antigen presentation [73]. They can also be utilized to enhance immune responses by providing sustained release capabilities [74].

Among the reviewed studies, most studies utilized encapsulated DNA molecules in CS for protection against bacteria (*A. hydrophila* and *P. damselae* subsp. *piscicida*) [75,76] or virus (SVCV) [77]. The results demonstrated the induction of both specific humoral and cellular immunity, as well as non-specific immune responses. Additionally, there was a significant increase in the expression of immune-related genes, antigen-specific IgM levels in serum, and leukocyte populations. Furthermore, improvements were observed in the vaccine’s mucoadhesivity and permeability, ultimately enhancing vaccine efficacy. Therefore, encapsulating vaccines in biocompatible carriers enhances their stability, targeted delivery, and immunogenicity, representing a promising strategy to improve disease control in aquaculture.

Beyond nucleic acid-based vaccine delivery, CSNPs and other polymers have also been explored as carriers for gene delivery and gene silencing in aquaculture species. NPs can protect DNA and RNA from degradation and facilitate their cellular uptake, intracellular release, and escape from lysosomal degradation, making it an attractive non-viral delivery system for genetic material [78,79]. Chitosan-based systems have been investigated for gene overexpression and gene silencing in aquaculture species [78] and other in vivo or in vitro models [80]. A particularly promising application in aquaculture is RNA interference (RNAi), in which double-stranded RNA (dsRNA) can be encapsulated in chitosan nanoparticles to improve its stability and delivery. Recent work has shown that chitosan nanoparticles can provide efficient dsRNA encapsulation and protection from RNase degradation, supporting their potential for oral delivery in shrimp [81,82]. This approach is particularly relevant for disease control because dsRNA can be designed to silence genes involved in viral infection, although challenges related to stability, release, tissue distribution, production costs, and scalability still need to be addressed [81,82]. A distinct and more advanced application is gene editing, where nanoparticle-based systems could potentially facilitate the delivery of CRISPR-associated components to modify endogenous genes. In aquaculture, gene editing has been proposed as a strategy to improve traits such as disease resistance and productivity, including in decapod species [83]. However, the application of nanoparticle-mediated gene editing remains largely prospective, and efficient delivery to the appropriate cells or tissues, adequate editing efficiency, control of off-target effects, and, where required, transmission of the modification to subsequent generations remain important challenges [83].

Nevertheless, the application of nanoparticle-mediated nucleic acid delivery approaches in aquaculture faces several technical, regulatory, and ethical challenges. These include limitations in achieving efficient, reproducible, and appropriately controlled delivery of genetic material in vivo [79], as well as regulatory and safety concerns associated with the administration of exogenous nucleic acids. Such concerns are not limited to genome-editing applications [84], but may also affect for example the development and approval of nucleic acid-based vaccines [85], particularly given the incomplete understanding of their potential long-term effects and the need to ensure their safety and efficacy. Consequently, this research line appears to have received considerably less attention in recent years. Despite these challenges, the available evidence highlights the potential of CSNPs as versatile carriers of genetic material for diverse applications in fish biotechnology.

##### Other Organic Nanoparticles

Similar to chitosan, other natural polymers have been tested as delivery systems (Table 2). Due to their sustained release properties, biodegradability, and biocompatibility, poly(lactic-co-glycolic acid) (PLGA)-based nanoparticles have become widely used in the development of polymeric drug delivery systems. By adjusting the monomer ratio, it is possible to control their loading capacity, degradation rate, and mechanical strength [86]. Harshitha et al. conducted two consecutive studies evaluating the use of PLGA-based nanoparticles for oral vaccination against *A. hydrophila* in zebrafish. In the first study, oral administration of a recombinant outer membrane protein A-based nanovaccine provided significant protection against *A. hydrophila* infection [87]. In the subsequent study, the recombinant maltoporin protein was encapsulated in PLGA nanoparticles and demonstrated potential as a vaccine candidate, further supporting the effectiveness of PLGA nanoparticle encapsulation for immunization purposes [88].

**Table 2:** Summary of agent delivery studies, detailing specific applications in aquaculture and their corresponding nanoparticle types based on the reviewed literature.

Application	Specific Effects	Type of NP	References
	An oral DNA nanovaccine was shown to effectively combat *Photobacterium damselae* subsp. *piscicida* in *Solea senegalensis* by activating specific humoral and cell-mediated immunity, as well as non-specific immune responses	Chitosan	[76]
	Following vaccination with an encapsulated DNA vaccine, SVCV-neutralizing antibody titers, serum antigen-specific IgM levels, and immune-related gene expression are markedly elevated in common carp infected with the virus	Mannosylated chitosan	[77]
	In zebrafish, a diet supplemented with a DNA vaccine incorporated into CS enhanced resistance to *A. hydrophila* infection by upregulating immune-related genes, preserving histological integrity, improving leukocyte populations, and promoting optimal tissue distribution	Chitosan-tripolyphosphate	[75]
	Oral administration of a recombinant outer membrane protein A-based nanovaccine conferred 77.7% relative survival in zebrafish challenged with *A. hydrophila*, while gene expression analyses revealed significant upregulation of immune-related genes in vaccinated fish	PLGA	[87]
	The results indicate that the vaccine embedded in nanoparticles induced higher levels of IFN1 and ISG expression in the intestine and head kidney of rainbow trout compared to fish fed with the vaccine alone	A type of phytoglycogen	[89]
Adjuvant of other components	Compared to the control group, a diet supplemented with N-acetyl-D-glucosamine-loaded CSNPs enhanced Nile tilapia’s specific growth rate, weight gain, survival rate, respiratory burst, and lysozyme activity	Chitosan	[70]
	The results showed that a chitosan-*Ocimum basilicum* nanocomposite dietary intervention can protect Nile tilapia from *Aeromonas sobria* and *C. albicans* infections while promoting growth, intestinal architecture, immunity, and antioxidant parameters	Chitosan	[69]
	The immune-stimulating and free radical–scavenging properties of selenium-loaded CSNPs against *A. hydrophila* contributed to the observed improvements in Nile tilapia growth	Chitosan	[67]
	To combat *S. agalactiae* infection, supplementing Nile tilapia diets with CSNPs containing vitamins C and E for 7 or 14 days prior to a salt-induced stress may enhance immune- and antioxidant-related gene expression	Chitosan	[66]
	When administered to grass carp, CSNPs loaded with *Ctenopharyngodon idella* interferon-γ2 led to elevated blood biochemical indices and increased expression of multiple immune-related genes	Carboxymethyl chitosan	[68]
	The findings showed that oral administration of chitosan combined with 100 μg GnRH markedly enhanced ovarian development and oocyte formation in goldfish	Chitosan	[71]
	Encapsulated green tea polyphenols exhibited in vitro activity against *Vibrio anguillarum*, *V. alginolyticus*, *Pseudomonas anguilliseptica*, and *S. iniae*, with particularly strong activity against *P. damselae*	Chitosan–alginate	[72]
	Enrofloxacin-loaded casein nanoparticles enhanced the absorption, prolonged the retention time and improved oral bioavailability of enrofloxacin	Casein	[90]
	In vitro, grape seed extract–loaded solid lipid NPs upregulated antioxidant genes and downregulated hsp70 and cytoskeleton-related genes, improving fish cell viability and antioxidant capacity	Lipids	[91]
	Lipid-based nanocarriers delivering an androgen hormone (17α-Methyltestosterone-Loaded Alkyl Polyglucosides) as a feed additive appear to be a viable and cost-effective approach to reduce the dosage required for masculinizing red tilapia	Lipids	[92]

Another promising yet underutilized group of polymers for vaccine delivery is natural polysaccharides. Samms et al. utilized synthetic double-stranded RNA embedded in phytoglycogen nanoparticles, which increased IFN1 levels and induced an antiviral state by upregulating interferon-stimulated genes (ISGs) in the intestine and head kidney of rainbow trout compared to fed controls [89].

In addition to the nanoparticles previously discussed, other organic nanoparticles have been investigated for drug delivery applications. For example, the limited use of enrofloxacin (ENR) in aquaculture, due to its poor water solubility and consequently low bioavailability, motivated studies by Yuan et al. conducted both in vitro and in vivo (in rats). They found that ENR-loaded casein nanoparticles enhanced drug absorption, prolonged retention time, and improved the oral bioavailability of ENR [90]. Regarding lipid-based carriers, Trapani et al. incorporated a natural antioxidant extract into solid lipid nanoparticles, which induced the overexpression of antioxidant genes and downregulation of hsp70 and other cytoskeleton-related genes in in vitro assays using sea bream (*Sparus aurata*) and European seabass (*Dicentrarchus labrax*) cell lines [91]. Furthermore, to reduce the dosage of synthetic sex-reversal hormones in aquaculture, Yostawonkul et al. employed solid lipid nanoparticles to deliver an androgen as a feed additive for on-farm masculinization of red tilapia [92].

From a translational perspective, the applicability of these organic nanoparticle systems depends not only on their biological efficacy but also on the simplicity of their preparation, loading capacity, scalability, and overall cost. These parameters vary substantially according to the nature of the carrier and the characteristics of the encapsulated molecule. For example, PLGA nanoparticles can be prepared using several techniques, including emulsion-based methods and nanoprecipitation, and their drug loading and release profiles can be modulated by adjusting polymer composition and formulation parameters [93]. However, transferring these processes from laboratory to industrial scale remains challenging because of batch-to-batch variability and the need for strict control of particle size, drug loading, encapsulation efficiency, and other critical quality attributes [93,94]. In contrast, solid lipid nanoparticles can be produced using relatively straightforward processes and have been considered particularly attractive from a manufacturing and scalability perspective [95]. The amount of active compound that can be incorporated is likewise highly dependent on its physicochemical properties and its compatibility with the nanoparticle matrix; therefore, high encapsulation efficiency does not necessarily translate into a high payload or efficient release of the biologically active compound. From an economic perspective, simpler formulations based on readily available natural polymers or lipids may offer advantages for large-scale aquaculture applications, whereas more complex synthetic polymeric systems may require greater investment in manufacturing, process optimization, and quality control. Consequently, although synthetic organic nanoparticles such as PLGA and lipid-based systems offer considerable flexibility for the delivery of vaccines and therapeutics, their practical implementation in aquaculture will depend on achieving a favourable balance between loading capacity, biological efficacy, manufacturing simplicity, scalability, and cost.

#### 3.1.3. Nanoparticles in Disease Diagnosis

In the aquaculture sector, beyond disease treatment interventions, it is essential to implement rigorous diagnostic monitoring to anticipate large-scale disease outbreaks. Key factors to monitor include the presence of environmental pathogens and metabolites associated with either the pathogen or infected fish.

Disease diagnosis is routinely performed using a variety of immunological and molecular methods [96]. For end-users, the effectiveness of these assays depends on factors such as cost, speed, specificity, and sensitivity. In this context, nanotechnology has emerged as a powerful diagnostic tool, offering high performance across all these parameters.

##### Gold NPs, the First Choice

Numerous nanoparticles have been applied in veterinary diagnostics, with noble metal nanoparticles, particularly gold nanoparticles (AuNPs), playing a prominent role as nanosensors, typically as part of diagnostic platforms. Their superior optical properties enable efficient detection without PCR amplification, providing high selectivity and sensitivity as colorimetric sensors, properties often associated with the spherical shape of AuNPs [97,98].

All studies reviewed over the past six years developed one-step prototype devices that reduce the cost of current techniques and enable rapid on-farm detection. A significant number of colorimetric assays utilizing functionalized AuNPs have been developed for detecting viruses relevant to aquaculture. These include assays for detecting the DNA of Largemouth Bass Virus (LMBV) [99], hybridization with whole virions of Nervous Necrosis Virus (NNV) in European seabass [100], and colorimetric assays based on reverse transcription loop-mediated isothermal amplification (RT-LAMP) to identify viral genomic segment 9 in Tilapia Lake Virus (TiLV) [101]. Aptasensor-based AuNP conjugates have been applied for the detection of non-fish viruses such as White Spot Syndrome Virus (WSSV) [102], while specific probes have been designed to detect bacterial toxin genes, such as pirAvp in acute hepatopancreatic necrosis disease (AHPND), with detection limits as low as 20 fg/µL [103]. Additionally, prototype devices targeting conserved RNA regions within the NNV capsid protein gene in sea bream and sea bass, based on amperometric responses, have achieved detection limits of 20 fM [104]. Colloidal gold immunochromatography specific for NNV has also been employed, with detection limits of approximately 10^3^ TCID_50_/mL and 10^5^ TCID_50_/100 μL, depending on the assay format [105,106].

For indirect monitoring in aquaculture, such as measuring creatinine and cortisol levels, non-invasive methods using AuNPs have been developed to detect them directly in water tanks. More sophisticated systems have also been employed, including hybrid fiber optic sensor probes based on localized surface plasmon resonance (LSPR) that incorporate AuNPs along with other signal-enhancing materials, such as MoS_2_ and Nb_2_CT_x_ [107,108,109].

Ultimately, the potential of these nanoparticles as biosensors allows them to serve as screening tools capable of determining whether a sample is positive or negative, as well as identifying samples with ambiguous disease status that require further laboratory analysis using alternative methodologies.

### 3.2. Water Monitoring and Treatment

**Figure 2:**
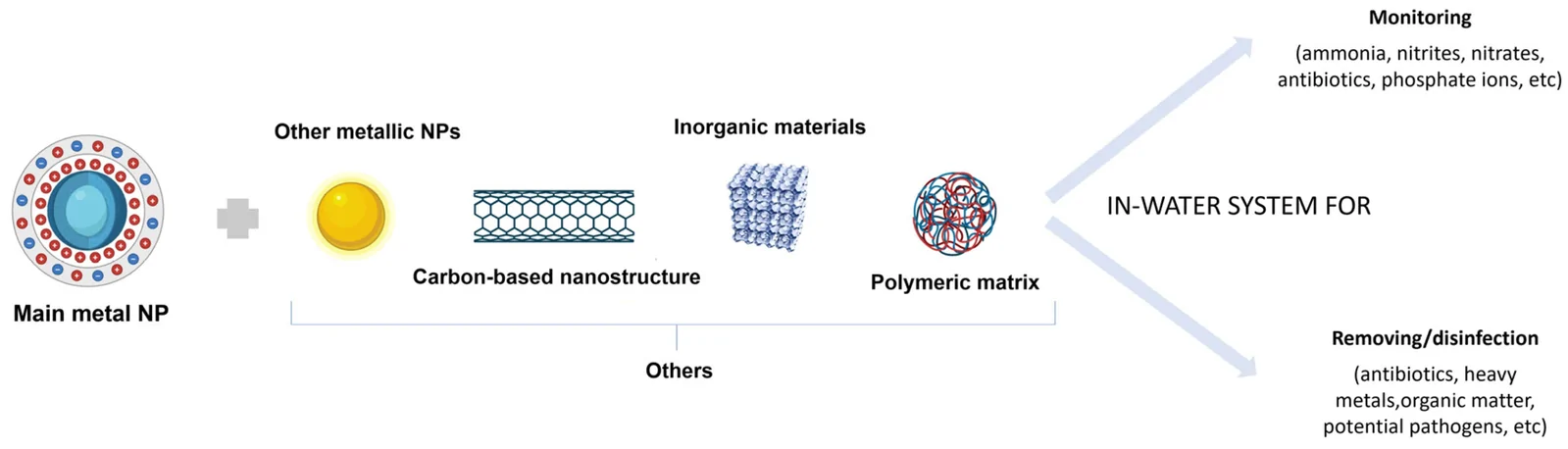
Common nanoparticle combinations for water treatment applications in aquaculture. Created in Biorender. Laura González-Rodríguez (2025) (https://biorender.com/).

Monitoring and treating the water is another type of action that can be carried out for both inland water and aquaculture plant effluents. This is necessary to ensure the wellbeing of the fish stored in the facility as well as to prevent additional environmental pollution. Nanoparticles can be used for three main purposes: (i) monitoring, (ii) removing contaminants, and (iii) disinfecting (Figure 2). Biological and organic compounds (including potential pathogenic microbes), heavy metals, and pesticide and pharmaceutical waste are just a few of the contaminants that stand out in this industry. Because they have a significant impact on the pond yield, water quality procedures also take into account the physicochemical parameters, including pH, dissolved oxygen (DO), biochemical oxygen demand (BOD), total dissolved solids (TDS), total suspended solids (TSS), chemical oxygen demand (COD), turbidity, water hardness, total organic matter (TOM), nitrate and phosphate content [110].

Conventional wastewater remediation relies on a combination of physical, chemical, and biological techniques. For example, solid waste is typically treated through mechanical filtration or sedimentation. However, micro- and nano-sized contaminants often persist after these procedures, limiting their overall effectiveness. Additionally, viruses, bacteria, and small protozoa are difficult to settle, requiring extra measures that increase costs [16]. Consequently, alternative approaches are needed, such as the use of NPs. Although research on their application has so far been limited to laboratory-scale studies, results are very promising. This review, therefore, focuses on the potential use of nanoparticles for maintaining water quality in aquaculture.

#### 3.2.1. Water Monitoring

In line with the diagnostic biosensors (Section 3.1.3), nanobiosensors have been proposed for environmental monitoring to detect various pollutants and water quality parameters; however, their application in aquaculture remains largely undeveloped [111]. In all studies reviewed in this context, the nanoparticles used are metallic and are typically incorporated into hybrid systems to enhance the sensitivity and selectivity of the resulting sensing platforms (Figure 2). Gold nanoparticles (AuNPs) remain the most widely employed in this setting. Accordingly, AuNPs have been investigated for the detection of nitrogen compounds, such as ammonia, nitrites, and nitrates, either as part of electrode systems or in combination with cyclodextrin or zinc [112,113,114]. Additionally, nanoparticles have been applied for the detection of antibiotic residues commonly used to combat bacterial infections, including norfloxacin and nitrofurazone [115,116].

Other metallic NPs, such as silver (Ag) or copper (Cu), have been used to detect nitrite, phosphate ions, and hydrogen peroxide [117,118,119], while iron (Fe), often incorporated into multifunctional complexes, has been employed for the detection, extraction, or degradation of antibiotics [120,121]. In some cases, nanoparticles are integrated into carbon-based structures [114,116,119] or polymeric networks [111], which provide chemical stability and enhanced adsorption capacity. Overall, these methods exhibit excellent limits of detection, ranging from 5.9 µM for nitrite [119] to 0.001 µM for antibiotics [120].

#### 3.2.2. Removal of Water Pollutants and Disinfection

Some research focuses exclusively on remediation, in addition to the previously proposed systems for detection and removal. Of particular importance is the development of nanoscale mixed-metal composites, whose constituents combine unique properties such as high adsorption capacity, a large surface area-to-volume ratio, and magnetic characteristics. These nanoparticles can be used to remove contaminants from water either through adsorption or by catalyzing their degradation [122]. Furthermore, certain metallic nanoparticles are effective at inactivating or trapping microorganisms in water due to their ability to induce non-visible membrane damage, alter bacterial cell membrane structure through internalization, and trigger apoptosis [123].

Among the works reviewed in this section, metallic nanoparticles such as iron (Fe), copper (Cu), zinc (Zn), silver (Ag), and titanium (Ti) are again the most commonly used. These are frequently combined with other metals [124,125,126], with carbon-based materials [124,125,127,128], with inorganic nanoparticles [129], or incorporated into polymeric matrices [130,131,132,133] (Figure 2).

Regarding the contaminants they can remove, the most prevalent nanoparticles target antibiotics commonly used in aquaculture, such as rifampicin, amoxicillin, and ciprofloxacin, among others [124,125,126,127,128,129,134,135]. Other studies focus on the removal of heavy metals, including iron, chromium, and cadmium [129,132,133,136,137], as well as phosphates and nitrogen residues [133,134,135,136,137,138,139]. Additional research has addressed the detoxification of other contaminants, such as organic matter [130] and pesticides and dyes [140].

In addition, several studies have assessed the disinfectant capacity of nanoparticles in conjunction with water remediation, demonstrating their ability to inactivate and/or remove bacteria such as *E. tarda*, *E. ictaluri*, *A. hydrophila*, *Enterococcus faecalis*, *E. coli* and other coliforms, *S. aureus*, *V. harveyi*, and certain *Pseudomonas* species [124,126,129,131,132,133,138]. Various approaches have been employed, including co-precipitation mechanisms that achieve complete bacterial removal even at high loads, composite filtration systems that reach over 99% efficiency, and photocatalysis, a widely used technique in the field [125,126,127]. The removal of potential pathogens is a critical component of water treatment protocols, both to maintain optimal health of cultured fish and to minimize the impact on human and animal health from effluents discharged from aquaculture facilities.

It is noteworthy that general studies on water disinfection over the past six years yield far more results than those specifically including ‘aquaculture.’ Thus, although the potential of nanoparticles for water remediation is well established, their application in aquaculture remains relatively recent.

#### 3.2.3. Limitations

Although nanomaterials have attracted growing attention as promising tools for wastewater treatment, their large-scale application is still limited by several technical and economic challenges that must be carefully evaluated. From an economic perspective, cost-effectiveness remains a major barrier to the adoption of nanomaterial-based water treatment technologies [141,142,143]. Consequently, increasing attention is being directed toward the development of cost-effective methods for nanoparticle synthesis. Moreover, regeneration and reusability of nanosorbents are critical for ensuring both the economic and environmental sustainability of these systems [141]. Several studies have highlighted that most nanosorbents exhibit reduced adsorption efficiency after multiple regeneration cycles, often due to nanoparticle agglomeration, changes in surface chemistry, or difficulties in recovering the particles from treated water [144]. Magnetically recoverable nanomaterials represent a promising approach, as the concentration of magnetic nanoadsorbents in treated effluents can be effectively controlled through magnetic separation, thereby reducing their potential ecotoxicological impact [145]. However, despite these advantages, their regeneration and reusability remain major challenges that hinder large-scale and long-term implementation [145].

### 3.3. Later Stages: Fish as a Product

Fish and shellfish are highly susceptible to deterioration due to enzymatic, chemical, and microbiological reactions, raising concerns about the safety of artificial preservatives currently used to maintain quality. Consequently, new approaches are continuously being explored, among which nanotechnology is particularly promising. Nanotechnology offers several advantages over conventional methods, including enhanced mechanical properties, improved heat resistance, and enhanced biodegradability.

Although nanoparticles are primarily used in food processing to enhance physicochemical properties and improve nutritional stability and bioavailability, the packaging phase is equally important for preserving these benefits. Notable applications of nanostructures include their use as antimicrobials, anti-caking agents, nanoadditives, nanocarriers, and nanosensors for monitoring food quality.

Although nanoparticles with such potential have been widely investigated across the food industry, their application in aquaculture remains relatively new, and few effective nanotechnology interventions have been implemented in this field to date [146,147]. Among the limited studies conducted during the period under review, those involving nanoparticles with antimicrobial properties are particularly notable. Incorporating these nanoparticles into packaging materials can extend the shelf life of fish products and reduce the risk of contamination.

Single zinc oxide (ZnO) nanoparticles have been shown to reduce bacterial and fungal populations when applied to packaging containers [148,149]. They can also be used as food additives to minimize lipid peroxidation, maintain pH levels, and enhance sensory qualities [144]. In another approach, ZnO nanoparticles are combined with Fe NPs to form a hybrid nanocomposite that preserves the product’s sensory attributes [150]. Additionally, antibacterial properties of Ag NPs have been validated at 4 °C and 8 °C [151]. These studies suggest that 3–7% of nanoparticles should be incorporated into packaging materials.

Nonetheless, it appears that more studies on the use of nanoparticles in final product packaging were conducted in the years prior to the period considered in this review. The reduction in studies during the reviewed period may be due to the fact that, although the potential applications of nanoparticles in fish packaging are promising, several important issues must be addressed. These include compliance with regulatory standards, evaluation of cost-effectiveness, and, most importantly, ensuring the safety of nanoparticle migration into food [152]. Ensuring consumer safety should remain the primary objective of research in this area.

### 3.4. Impact of NPs at Multiple Levels: Fish, Consumer, and Environment

Although nanoparticles hold significant potential in aquaculture, their potential dispersion across multiple levels has raised concerns regarding biosafety. Consequently, evaluating and characterizing the possible risks associated with their use is essential, with particular emphasis on understanding the prospective effects of nanoparticles on both target and non-target organisms.

The potential hazards of nanoparticles are influenced by various physical and chemical characteristics, including size, shape, surface coating or modifications, solubility, and chemical composition, which collectively determine their interactions with the environment [153]. For example, as nanoparticles decrease in size within the nanometer range, their intrinsic toxicity increases. Metals such as copper, silver, gold, and aluminium, while hazardous at high concentrations, can exhibit heightened toxicity at smaller particle sizes. Additionally, long-term exposure to nanoparticles may pose risks to both the environment and human health. Therefore, further research is necessary to establish safe and effective application levels that take into account these physicochemical properties.

Given their expected large-scale production and use in aquaculture, nanoparticles are likely to be released into the environment. The behavior of these released particles may change after fulfilling their intended purpose and can further fluctuate through interactions with natural ecosystem components, making it difficult to accurately assess their long-term environmental fate and impact [154]. Moreover, while potential environmental effects may be partially mitigated by using efficient filtration and containment systems, the physiological effects of NPs on cultured species remain more challenging to control.

Several studies have evaluated these effects through exposure experiments, showing that nanoparticles can enter aquatic organisms either unintentionally (e.g., via contaminated water) or intentionally (e.g., through dietary supplementation). For instance, incorporating chitosan nanoparticles (CSNPs) into Nile tilapia diets has been reported to be safe, improving hematological parameters and intestinal morphology without compromising immunity or growth [155]. In contrast, polycaprolactone (PCL) nanoparticle-based encapsulation systems have shown even higher safety levels in zebrafish [156]. However, exposure to metallic nanoparticles has often revealed significant toxic effects. Studies in *Clarias gariepinus* and *Poecilia sphenops* exposed to AgNPs demonstrated alterations in behavior, hematological indices, neurological function, and reproductive parameters, resulting in acute toxicity [157,158]. Similarly, sublethal exposure of goldfish to CuNPs led to pronounced changes in blood biochemistry—such as reduced leukocyte and glucose levels and increased protein and lipid concentrations—alongside tissue injury, suggesting dose-dependent toxicity [159]. In the case of ZnNPs, chronic exposure in goldfish caused only mild histopathological alterations, though previous research has described oxidative and inflammatory effects in endothelial tissues [160].

Along the aquaculture production chain, potential consumer exposure should also be considered, either through ingestion of nanoparticle-enriched products or migration of particles from food packaging. Ingestion, together with inhalation and dermal absorption, constitutes one of the primary routes of nanoparticle entry into the human body. Nanoparticles can cross the gastrointestinal barrier via multiple mechanisms, including transcellular uptake by enterocytes, M-cell transport, persorption, and paracellular diffusion. Once absorbed, they can circulate through the bloodstream and accumulate in organs, such as the liver [153,161]. In vitro studies have demonstrated that ingested nanoparticles can induce oxidative stress and protein denaturation due to excessive ROS generation [147].

Recent assessments have addressed the potential for nanoparticle bioaccumulation along the food chain. The escalating use of inorganic nanoparticles such as TiO_2_ and Ag in aquaculture has raised concerns about their environmental release and trophic transfer. A recent study evaluated the bioaccumulation of these nanoparticles in sea bream, sea bass, and Japanese carpet shell following dietary exposure (0.25–1.5 mg kg^−1^ for up to 90 days). Moderate accumulation was detected in non-edible tissues (liver and kidney) [154], whereas muscle tissue showed no significant nanoparticle retention even at high exposure levels. Conversely, considerable accumulation of both TiO_2_ and AgNPs was observed in the soft tissues of the Japanese carpet shell. In vitro assays using the Caco-2 human intestinal model further indicated limited intestinal transport (<34% for TiO_2_ in sea bream, <61% and 4% for TiO_2_ and Ag in Japanese carpet shell, respectively). These results suggest that although bioaccumulation may occur in certain species and tissues, the risk to human consumers appears to be low due to limited nanoparticle uptake from edible portions [154].

Nevertheless, long-term and chronic exposure scenarios remain insufficiently understood. Therefore, before their broad implementation in aquaculture, a comprehensive toxicological evaluation of each nanoparticle type is necessary, considering key parameters such as temperature, pH, and salinity. In this regard, regulatory agencies such as the U.S. Food and Drug Administration (FDA) and the UK Food Standards Agency (FSA) have emphasized the need for rigorous safety assessments prior to authorizing the use of nanotechnology in food or aquaculture-related applications [5].

## 4. Conclusions

In summary, nanotechnology represents a promising tool to address longstanding challenges in the aquaculture industry, thereby improving current practices. However, the study of nanosafety has received comparatively less attention than the rapid expansion of research on nanoparticles and their potential applications. Consequently, it is advisable to test the specific nanoparticles or combinations intended for use in a particular project, rather than relying solely on emerging regulatory standards.

In accordance with the guidelines of the European Food Safety Authority (EFSA) and with the goal of expanding available data on nanoparticle toxicity, it is now possible to assess potential risks through the proposed evaluation of nanomaterials. This assessment includes various tests to evaluate nanotoxicity, such as bioaccessibility and bioavailability, among others. Basudan (2026) for instance, proposed a series of tests beginning with in vitro screening to examine excretion, metabolism, distribution, and absorption of nanoparticles at the cellular and intracellular levels, complemented by detailed physicochemical analyses and in vivo assays to evaluate systemic toxicity as well as cardiovascular, reproductive, neurological, and immunological effects [153]. Additionally, Akhtar et al. (2025) recommend the use of in silico techniques to predict the nanotoxicity of specific nanostructures, which can either supplement or replace some costly and time-consuming tests, particularly in the early stages of nanomaterial design [161]. Consequently, the number of studies evaluating the safety of nanoparticle applications should be proportionate to the growing body of research investigating their potential benefits.

## Data Availability

No new data were created or analyzed in this theoretical manuscript. Data sharing is not applicable to this article.
